# Effective handling of substantial arterial air embolization during extracorporeal perfusion

**DOI:** 10.1002/ccr3.2510

**Published:** 2019-11-24

**Authors:** Mohammad Bashar Izzat

**Affiliations:** ^1^ Department of Surgery Faculty of Medicine Damascus University Damascus Syria

**Keywords:** air embolism, cardiopulmonary bypass, heart, surgery

## Abstract

This report highlights the need for a coordinated approach to substantial arterial air embolization, considering the high risk of neurologic injury. Appropriate management may involve systemic hypothermia, hyperoxia, and retrograde cerebral perfusion.

## 1 INTRODUCTION

The occurrence of substantial arterial air embolization during open‐heart procedures is a rare incident, but which can result in direct occlusion of cerebral arteries and serious neurological consequences.^1^, ^2^ Coordination and decisiveness in the responses of the operating team can play a major role in preventing or at least minimizing damage that may result from such a critical event.

We report a case of substantial arterial air embolization which occurred during extracorporeal perfusion, and which we treated successfully with a combination of systemic hypothermia, hyperoxia, and retrograde cerebral perfusion.

## 2 CASE REPORT

A 67‐year‐old lady with symptomatic multivessel coronary artery disease was admitted to our service. Due to the presence of severe left main stem lesion, she underwent elective on‐pump coronary artery bypass grafting. The heart‐lung machine in use was a Stöckert S5 perfusion system with a blood level sensor (LivaNova PLC), supplied with an Inspire 8 oxygenator and a D734 Micro 40 arterial line filter (Sorin). All equipments had undergone standard preoperative check confirming ordered performance.

After completion of coronary grafting and as the patient was being weaned off‐bypass, the blood level inside the reservoir suddenly went down drastically, but the level sensor failed to trigger a forced pump‐head stop. The reservoir was drained out of blood, and a large bolus of air was pushed through the arterial line. Promptly recognized by the surgeon, the perfusionist was directed to turnoff the pump instantly, and the patient was placed in a steep Trendelenburg position. The arterial cannula was disconnected from the arterial line and the heart was massaged forcefully, and this purged a large amount of air out of the aorta. At the same time, the arterial line was connected to the venous line, and the pump head was operated to refill the arterial line with blood, driving all visible air out of the circuit. The arterial cannula was then reconnected to the arterial line, and extracorporeal perfusion was resumed, employing 100% inspired fraction of oxygen and maintaining a high mean arterial pressure (˃100 mm Hg). Dexamethasone (24 mg) and sodium pentobarbital (1.0 g) were administered for cerebral protection at the same time.

Systemic hypothermia was induced rapidly and, upon reaching a systemic temperature of 18°C, extracorporeal perfusion was halted, and retrograde cerebral perfusion with 500 mL/min of cold blood was carried out through the superior vena cava to flush out residual air from the cerebral arteries. The central venous pressure was constantly maintained below 30 mm Hg. This was continued for 30 minutes, and by the end of this period vented blood from the aortic root was free of any perceptible air. Extracorporeal perfusion was then resumed with slow systemic rewarming until normothermia was reached (total extracorporeal perfusion time 196 minutes). Electrocardiography did not show any changes or arrhythmias which might point to coronary artery embolization, and the patient was weaned easily from bypass with no inotropic support.

Through the initial 48 hours following surgery, the patient was held in a 30‐degree upright position, and mean arterial pressure was maintained >100 mm Hg. Other neuroprotective strategies included ventilation with 100% inspired fraction of oxygen and maintenance of normoglycemia, normal serum electrolytes, and slight hypocapnia. Pharmacological agents used for neuroprotection included dexamethasone and mannitol. Despite withholding all sedatives, the patient regained a diminished level of consciousness slowly, but there were no focal motor deficits. Computed tomography of the brain was performed on the 3rd postoperative day and showed normal differentiation of the cortex and the medulla, with no signs of intracranial bleeding or displacement of brain substance. The patient improved gradually over a period of 4 days, achieving complete neurological recovery and satisfactory respiratory effort by day 4, and was subsequently extubated. On the 14th postoperative day, she was discharged from hospital with no neurological defects.

## 3 DISCUSSION

Most frequently reported causes for substantial arterial air embolization during extracorporeal perfusion include sudden reduction in venous return to the blood reservoir, faulty reversal of cardioplegia or vent lines, inadequate clearance of intracardiac air, and oxygenator defects.^2^, ^3^ Air bubbles in the cerebral arteries can cause immediate mechanical obstruction to blood flow and direct endothelial damage, followed by an inflammatory response that damages the blood‐brain barrier and leads to cerebral edema, neuronal apoptosis, and serious neurologic sequelae.^4^, ^5^ Large bubbles generally pose a much greater threat to blood flow because they obstruct larger proportions of the vascular tree and take longer to dissolve completely.^1^

Numerous intra‐ and postoperative maneuvers have been described for managing substantial arterial air embolization, yet none have definitively been shown to be neuroprotective. These included systemic cooling, hyperoxia, retrograde cerebral perfusion, and hyperbaric oxygen therapy.^2^, ^3^, ^4^, ^5^, ^6^, ^7^

Hypothermic extracorporeal perfusion has been shown to be neuroprotective,^2^ and simultaneous use of a pH‐stat protocol can further increase cerebral blood flow and expedite brain cooling.^2^ Lowering blood temperature also increases gas solubility and promote rapid absorption of residual air emboli within the cerebral arteries. The additional use of a high inspired oxygen fraction decreases the partial pressure of nitrogen and affords an increased pressure gradient for accelerated nitrogen absorption from bubbles.^8^

Several authors have reported using retrograde cerebral perfusion successfully in cases of cerebral air embolization.^2^, ^6^, ^7^ Yet, no definitive perfusion or cooling protocols in this setting have been established. It is likely that the duration of retrograde cerebral perfusion should be limited to few minutes only in normothermic patient in order to avoid warm ischemia. Based on collective experience in aortic arch surgery,^9^ we decided to cool this patient to 18°C and to carry out retrograde cerebral perfusion with 500 mL/min of cold blood for 30 minutes, presuming that this may protect from ongoing cerebral insult.

Even though hyperbaric oxygen therapy has been shown to be effective in managing gross arterial air embolization,^4^, ^7^, ^10^ such service was not available in any nearby institution in the Middle East. Hyperbaric oxygen therapy has been most effective when initiated within hours of the incident, but this commonly requires transferring the patient to other medical facilities, with the associated limitations with monitoring and treating of the often‐unstable patient.

A final critical point is that, when substantial arterial air embolization is confronted, a coordinated and decisive approach is warranted to prevent or attenuate potential neurologic injuries.^3^ The increasing rarity of such a critical event can lead to an inadequate response by the surgical team, and this calls for establishing a set management protocol, and we believe that this successful case may be useful in this setting.

## CONFLICT OF INTEREST

None declared.

## AUTHOR CONTRIBUTIONS

Mohammad Bashar Izzat: involved in conception and acquisition of data, drafting the manuscript and revising it critically, and giving final approval of the version to be published.

## ETHICAL APPROVAL

All procedures performed in this study were in accordance with the ethical standards of the Damascus University Research Ethics Committee and with the 1964 Helsinki Declaration and its later amendments.

## INFORMED CONSENT

Informed consent was obtained from the participant included in the study.
